# Proteome-wide Analysis of Lysine 2-hydroxyisobutyrylation in Developing Rice (*Oryza sativa*) Seeds

**DOI:** 10.1038/s41598-017-17756-6

**Published:** 2017-12-13

**Authors:** Xiaoxi Meng, Shihai Xing, Loida M. Perez, Xiaojun Peng, Qingyong Zhao, Edilberto D. Redoña, Cailin Wang, Zhaohua Peng

**Affiliations:** 1 Department of Biochemistry, Molecular Biology, Entomology and Plant Pathology, Mississippi State University, Starkville, Mississippi 39762 USA; 2 Department of Bioinformatics, Jingjie PTM Biolab Co. Ltd, Hangzhou, 310018 China; 3 Institute of Crop Sciences, Jiangsu Academy of Agricultural Sciences, Nanjing, 210014 China; 4 Delta Research and Extension Center, Stoneville, P.O. Box 197, Mississippi, 38776 USA; 5 Present Address: Institute of Traditional Chinese Medicine Resources Protection and Development, Anhui Academy of Chinese Medicine, Hefei, Anhui 230000 China

## Abstract

Lysine 2-hydroxyisobutyrylation is a recently identified protein post-translational modification that is known to affect the association between histone and DNA. However, non-histone protein lysine 2-hydroxyisobutyrylation remains largely unexplored. Utilizing antibody-based affinity enrichment and nano-HPLC/MS/MS analyses of 2-hydroxyisobutyrylation peptides, we efficaciously identified 9,916 2-hydroxyisobutyryl lysine sites on 2,512 proteins in developing rice seeds, representing the first lysine 2-hydroxyisobutyrylome dataset in plants. Functional annotation analyses indicated that a wide variety of vital biological processes were preferably targeted by lysine 2-hydroxyisobutyrylation, including glycolysis/gluconeogenesis, TCA cycle, starch biosynthesis, lipid metabolism, protein biosynthesis and processing. Our finding showed that 2-hydroxyisobutyrylated histone sites were conserved across plants, human, and mouse. A number of 2-hydroxyisobutyryl sites were shared with other lysine acylations in both histone and non-histone proteins. Comprehensive analysis of the lysine 2-hydroxyisobutyrylation sites illustrated that the modification sites were highly sequence specific with distinct motifs, and they had less surface accessibility than other lysine residues in the protein. Overall, our study provides the first systematic analysis of lysine 2-hydroxyisobutyrylation proteome in plants, and it serves as an important resource for future investigations of the regulatory mechanisms and functions of lysine 2-hydroxyisobutyrylation.

## Introduction

Post-translational modifications (PTMs) play a key role in the regulation of protein functions. A wide array of novel protein PTMs has been discovered throughout recent years including crotonylation, propionylation, butyrylation, biotinylation, glutarylation, succinylation, malonylation, and ADP ribosylation^1^. Many of these PTMs have been reported to modulate chromatin packaging and DNA-binding proteins accessibility by either altering the charge state of histones or through internucleosomal interactions^1^. In addition, PTMs may have impacts on associations of regulating enzymes or protein effectors to the modified substrates, thus further affecting protein functions^2^.

**Figure 1:**
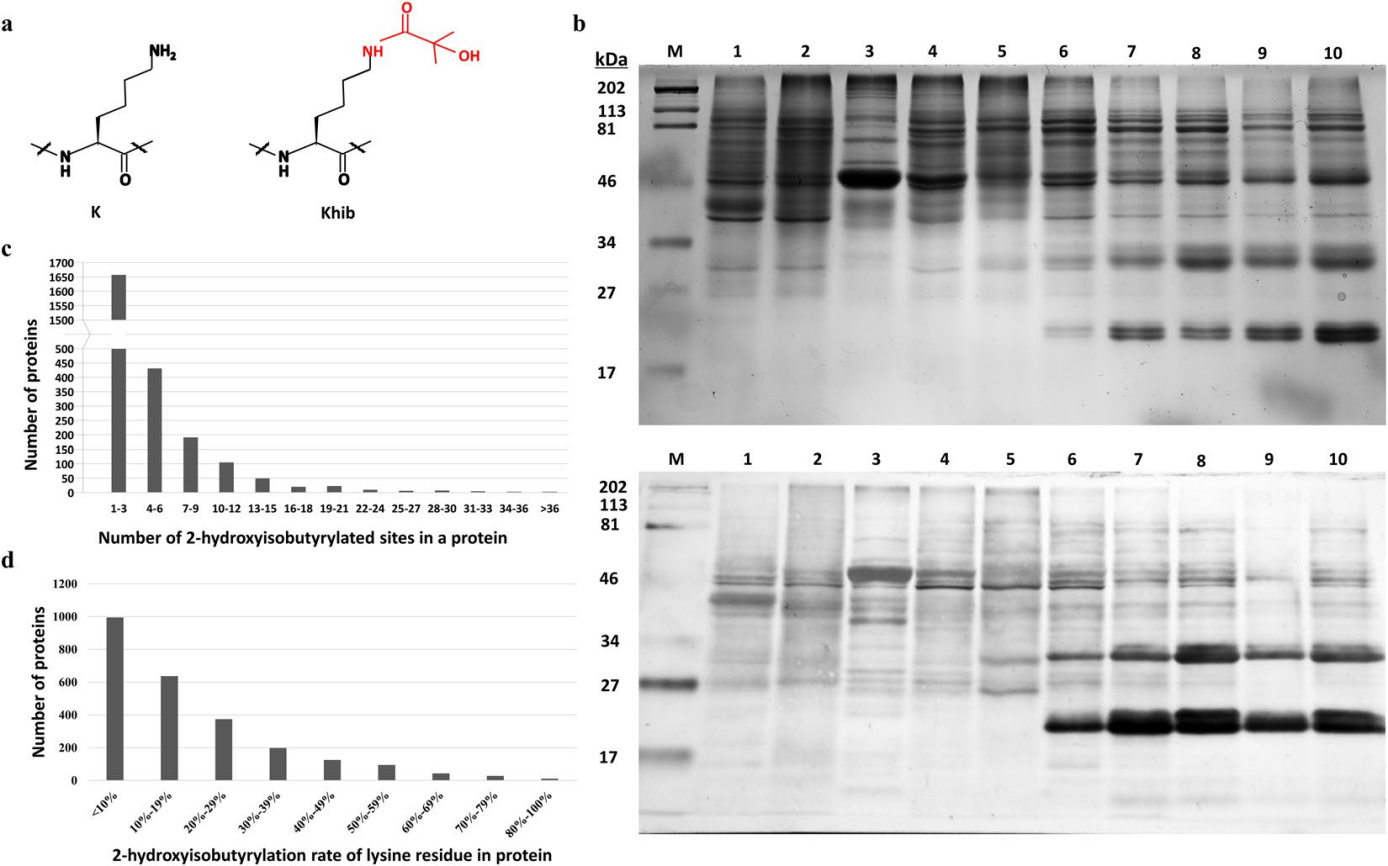
Lysine 2-hydroxyisobutyrylation in different rice tissues and proteins. (**a**) Structure of lysine 2-hydroxyisobutyrylation. (**b**) Lysine 2-hydroxyisobutyrylation profile in different rice organs/tissue revealed by western blotting. Molecular weight is labelled on the left. The samples are labelled on the top. M: size marker; 1. suspension cell protein; 2: roots protein; 3: leaves protein; 4: flowers protein; 5: pollens protein; 6: protein from 7 dpa seeds; 7: protein from 15 dpa seeds; 8: protein from 21 dpa seeds; 9: protein from mature dry seeds; 10: protein from mature dry seeds. Upper: Image of SDS-PAGE stained with coomassie blue. Lower: western blotting image. Same amount of proteins (25 μg per lane) were loaded for sample 1–8 and 10; 20 μg protein was loaded for sample 9. The original images of SDS-PAGE and western blotting are shown in Supplementary Fig. S1. (**c**) Distribution of 2-hydroxyisobutyrylated proteins based on number of modification sites. (**d**) Distribution of 2-hydroxyisobutyrylation rate on lysine residues of 2-hydroxyisobutyl-proteins.

In recent investigations, Zhao and his co-workers discovered the innovative, evolutionarily-conserved short-chain lysine acylation, 2-hydroxyisobutyrylation (K_hib_)^3^. K_hib_ significantly introduces a steric bulk with a mass shift of + 86.03 Da due to acylation of the epsilon amino side-chain of lysine (Fig. 1a). 2-hydroxyisobutyryl group accumulation neutralizes lysine’s positive charge, potentially fluctuating the association between histone and DNA. Concurrently, it attaches a hydroxyl group to the designated substrate to enable hydrogen bond formation between the modified lysine and other molecules^3,4^. Moreover, K_hib_ is associated with active gene transcription and H4K8hib is a better indicator for high gene expression than H4K8ac^3,5,6^. Isotopic labeling of core histones from HeLa cells showed that 2-hydroxyisobutyryl-CoA (HibCoA), that can be synthesized from 2-hydroxyisobutyrate, is likely a cofactor for K_hib_^3,5,6^. Histone acetyltransferase Esa1p in budding yeast and its homologue TIP60 in human could trigger K_hib_ reaction both *in vitro* and *in vivo* ^7^. In addition, *in vitro* experiments provided noteworthy evidence of histone deacetylases 1–3 (HDAC 1–3), Rpd3p, and Hos3p functioning as potential regulatory enzymes for lysine de-2-hydroisobutyrylation reactions on core histones^3,8^.

Presently, 63 histone 2-hydroxyisobutyrylated lysine sites have been identified in human and mouse^3^, which exceeds the known numbers of other extensively-studied histone PTM sites. Notably, 27 of these 63 modified sites are exclusive to 2-hydroxyisobutyrylation compared to lysine acetylation and crotonylation^3^. The stoichiometries of histones H3K79, H2BK108, H4K91 and H1K62 in synchronized G2/M Hela cells were reported as comparable to or even higher than that of many histone acetylation marks with known biological functions, indicating that K_hib_ is a highly dynamic PTM in cell^1,3^. Zhao *et al*. (2014) revealed that K_hib_ positioned in both N termini and main globular domains of histones encounters a modification mechanism that differs from acetylation^3^. In addition, K_hib_ has shown dramatic changes in genomic distribution during male germ cell differentiation^3^. Past investigations specified that histone K_hib_ level can be globally regulated by glycolytic rate. Unlike lysine acetylation, it showed a quantitative response to changes in glycolytic flux^6^. These findings suggest that lysine 2-hydroxyisobutyrylation is structurally and mechanistically distinct from lysine acetylation^3^. Xiao *et al*. (2015) developed the method for site-specific incorporation of ε-N-2-Hydroxyisobutyryl-lysine into bacteria and mammalian cells by utilizing the amber suppression-mediated strategy^4^, providing a beneficial tool for investigation of 2-hydroxyisobutyrylation’s biological functions.

Lysine 2-hydroxyisobutyrylation signals have been detected in HeLa cells, mouse embryonic fibroblast (MEF) cells, *Drosophila* S2 cells and yeast *Saccharomyces cerevisiae* cells on both histone and non-histone proteins by western blotting^3^. These suggest that K_hib_ should be present in non-histone proteins and should have functions independent of nucleosomes. Proteome-wide profiling of K_hib_ reported an identification of 6,548 K_hib_ sites on 1,725 substrate proteins in mammalian cells^7^. HPLC-MS/MS-based proteome analysis in *Saccharomyces cerevisiae* identified 1,458 K_hib_ sites on 369 proteins, and further bioinformatics analysis revealed that K_hib_ is enriched in the glycolysis/gluconeogenesis pathway^8^. Detection of the non-histone substrates and modification sites is necessary for additional characterization of the functions and regulatory mechanisms governed by lysine 2-hydroxyisobutyrylation. However, non-histone substrates of lysine 2-hydroxyisobutyrylation have not been reported in plants thus far.

Rice (*Oryza sativa*) is the staple food for over half of the world’s population, and it is crucial for global food security^9^. The regulations and mechanisms of grain filling and nutritional accumulation during rice seed development have garnered interest because of the magnitude of their impact in overall human health, food security, grain yield, and economic value. Moreover, 2-hydroxyisobutyrylation blocks trypsin cleavage at lysine residues that may impact the ability to digest and absorb cereal proteins in human body. In order to explore the K_hib_ status in rice proteome and the potential regulatory functions of K_hib_ for grain filling and development, we provide the initial identification and characterization of lysine 2-hydroxyisobutyrylome in developing rice seeds. Our study revealed that lysine 2-hydroxyisobutyrylation is broadly obtainable in the plant cells. The modification is highly conserved and enriched in a number of important biological processes. Lysines in both histone and non-histone proteins can be co-modified by K_hib_ and other acylations. These findings have broadened our perception of lysine 2-hydroxyisobutyrylation involvement in protein regulation, and they will be instrumental for illustrations of the potential functions and regulatory metabolisms of this newly identified PTM.

## Results and Discussion

### Identification of lysine 2-hydroxyisobutyrylation modification in developing rice seeds

To detect the 2-hydroxyisobutyrylation status in rice, proteins isolated from rice suspension cells, roots, leaves, flowers, pollens, as well as 7 dpa, 15 dpa, 21 dpa and mature seeds were examined by western blotting using pan anti-2-hydroxyisobutyryllysine antibody (Fig. 1b and Supplementary Fig. S1). Multiple protein bands of different sizes and smears were observed from all tested samples in western blotting, which displayed a pattern different from the one revealed by SDS-PAGE, suggesting that protein K_hib_ is ubiquitous in rice cells and specific for selected proteins.

We performed proteome-wide lysine 2-hydroxyisobutyrylaton identification in 15 dpa developing rice seeds. Following tryptic digestion, affinity enrichment and nano-HPLC/MS/MS analysis, a total of 9,916 2-hydroxyisobutyryl lysine sites across 2,512 proteins were identified with false discovery rate (FDR) of ≤1%. Detailed information of all identified 2-hydroxyisobutyryl peptides are shown in Supplementary Table S1. The mass spectrometry proteomics data have been uploaded onto the ProteomeXchange Consortium^10^ via the PRIDE partner^11^ repository with the dataset identifier of PXD005986. The average peptide score was 101.0119 and the average modified peptide length was 15 amino acids (see Supplementary Fig. S2). The majority of the 2-hydroxyisobutyryl proteins carried 1 to 3 K_hib_ sites, while around 9.2% proteins carried more than 10 K_hib_ sites (Fig. 1c). To evaluate the coverage of K_hib_ in substrate protein, the rate of 2-hydroxyisobutyrylated lysines out of total lysine residues in the each modified protein was counted and showed in Fig. 1d. Most identified proteins had less than 20% lysines that harbor 2-hydroxyisobutyrylation. Notably, 180 proteins had 2-hydroxyisobutyrylated lysine ratio over 50%. The rice 2-hydroxyisobutyrylome is larger than the reported acetylome, succinylome and ubiquitome in plants, which is consistent with previous reports that more K_hib_ sites are present on histones than any other PTM sites^12–14^, suggesting that lysine 2-hydroxyisobutyrylaton is an abundant PTM and may have more profound roles on substrate protein regulations in plants.

### Analysis of 2-hydroxyisobutyrylated sites

**Figure 2:**
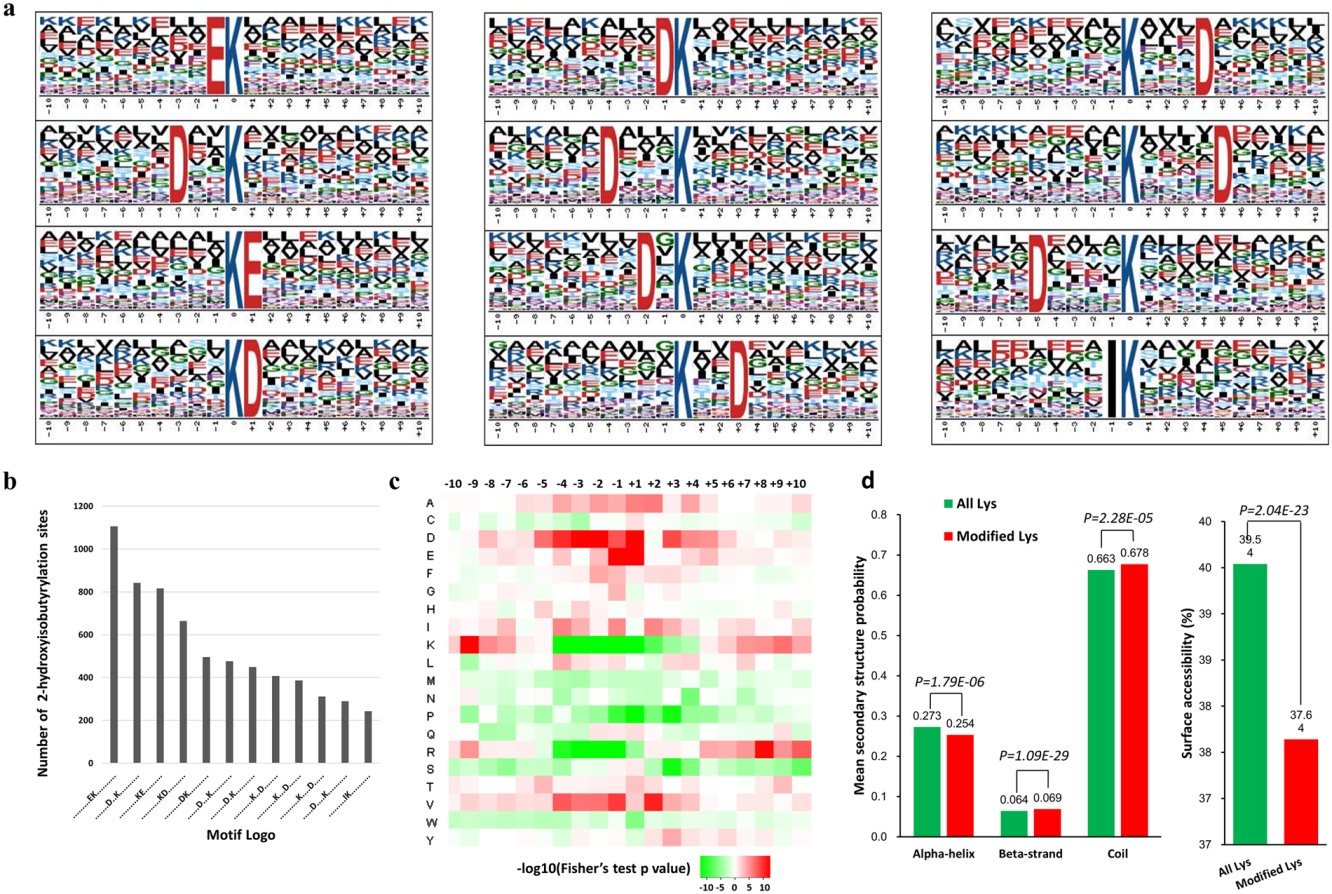
Characteristics of the 2-hydroxyisobutyrylated sites. (**a**) Conserved motifs of 2-hydroxyisobutyryl-21-mers flanking the modification sites (‘K’ in position 0). The size of each letter correlates to the frequency of that amino acid residue occurring in that position. (**b**) Frequency of each motif occurred in the identified 2-hydroxyisobutyrylated peptides. (**c**) Heat map of the amino acid compositions around the 2-hydroxyisobutyrylation sites. The –log10 (Fisher’s exact test p value) for every amino acid in each position (from −10 to 10) is shown. (**d**) Secondary structure and surface accessibility analyses of 2-hydroxyisobutyrylated lysine residues.

Specific amino acid sequence motifs around the 2-hydroxyisobutyrylated sites were detected by Motif-x tool. Twelve motifs were identified for amino acid residues located within 10 amino acids upstream and downstream (2-hydroxyisobutyryl-21-mers) of the 2-hydroxyisobutyrylated lysines (Fig. 2a). The results revealed a strong bias for negatively charged side chain amino acids, aspartic acid (D) and glutamic acid (E), around the modified lysine residues. The three most enriched motifs with 1106, 842, and 816 matches were [EK_hib_], [D_XX_K_hib_], and [K_hib_E], respectively (K_hib_ indicates the 2-hydroxyisobutyrylated lysine, and x indicates a random amino acid residue, Fig. 2b). Furthermore, the frequency of amino acids flanking the 2-hydroxyisobutyrylated lysine site was analyzed (Fig. 2c). Besides D and E, lysine (K) at −9 position, arginine (R) at +8 position, and valine (V) at −1 and +2 positions were over-presented around the modified lysine sites. However, lysine (K), proline (P), arginine (R) and serine (S) at position −4 to +4, were under-presented around the 2-hydroxyisobutyrylated lysines (Fig. 2c). The amino acid sequence features around the modification sites are usually determined by the specificity of catalytic enzymes. Experiments on core histones found that histone deacetylases HDAC1-3 are potential enzymes to regulate lysine de-2-hydroxyisobutyrylation *in vitro* ^3^. Similar to 2-hydroxyisobutyrylation, the preference of amino acid D and E flanking the modified lysine residue was also observed from acetylomes of rat, *M*. *abscessus* and *T*. *thermophilus* ^15–17^, although at a less prevalent level and the precise position of D and E are different. The similar amino acid sequence feature around the modification sites detected from both 2-hydroxyisobutyrylome and acetylomes may explain the functions of HDAC1-3 on de-2-hydroxyisobutyrylation.

To determine the preferred structure of the K_hib_ site in proteins, secondary structure analysis was performed using NetSurfP (Fig. 2d). Approximately 32.3% of the 2-hydroxyisobutyrylated lysines were located at regions of ordered secondary structure (alpha-helix and beta-strand), while the majority of K_hib_ sites occur within regions of intrinsic disorder and coil region. Moreover, K_hib_ tended to occur moderately in beta-strands and coil regions when compared to unmodified lysine residues. It is suggested that the flexibility of disordered regions allows the modified amino acid residue to be located in a folded polypeptide chain. Therefore, the amino acid side chains would easily fit into a modifying enzyme’s catalytic site^18^. Additionally, lysine residues at K_hib_ sites were less surface-accessible compared with unmodified counterparts (Fig. 2d). Non-specific modified lysines would have a higher likelihood to distribute evenly in protein and/or with higher surface accessibility. The high sequence specificity revealed by modification site motif analysis and lower surface accessibly of the K_hib_ sites suggested that lysine 2-hydroxyisobutyrylation occurs in a selective process.

### Functional enrichment analyses and cellular localization of 2-hydroxyisobutyrylated proteins

**Figure 3:**
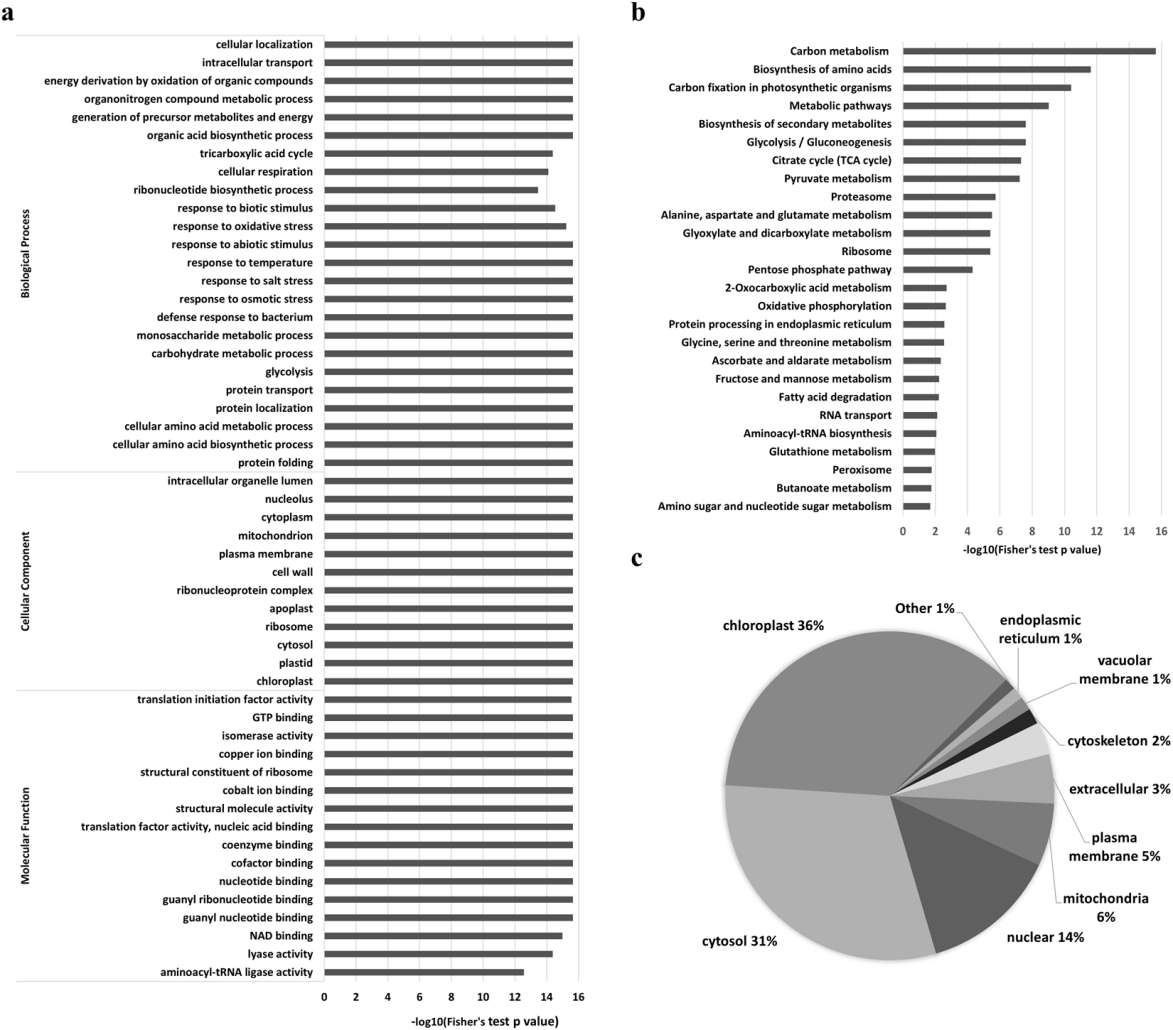
Enrichment and subcellular localization analyses of 2-hydroxyisobutyrylated proteins. (**a**) GO-based enrichment analysis of identified proteins. The value of -log10 (Fisher’s test p value) is shown. (**b**) KEGG pathway enrichment analysis of identified proteins. The value of -log10 (Fisher’s test p value) is shown. (**c**) Subcellular localization of the identified 2-hydroxyisobutyrylated proteins.

Functional enrichment analyses of GO and KEGG pathway revealed that 2-hydroxyisobutyrylated proteins in developing rice seeds were involved in multiple pivotal metabolic processes (Fig. 3a and Supplementary Table S2).

Enrichment analysis of GO biological process demonstrated that 2-hydroxyisobutyrylated proteins associated with responses to biotic and abiotic stimulus, including responses to bacterium, oxidative stress, temperature, salt stress and osmotic stress, were significantly enriched in 2-hydroxyisobutyrylome (Fig. 3a), indicating that K_hib_ may play important roles in plant adaption to different environment stimulus. In addition, proteins involved in amino acid metabolic processes and biosynthesis, as well as protein folding, transport and localization were enriched in 2-hydroxyisobutyrylome of the developing rice seeds (Fig. 3a), suggesting a potential role of K_hib_ in protein biosynthesis and processing. Enrichment analysis of GO molecular function demonstrated that proteins bearing K_hib_ were significantly related to enzyme activities and binding of various targets (Fig. 3a). Consistent with biological process enrichment analysis, proteins involved in translation and aminoacyl-tRNA ligase activities were highly enriched with 2-hydroxyisobutyrylation.

**Figure 4:**
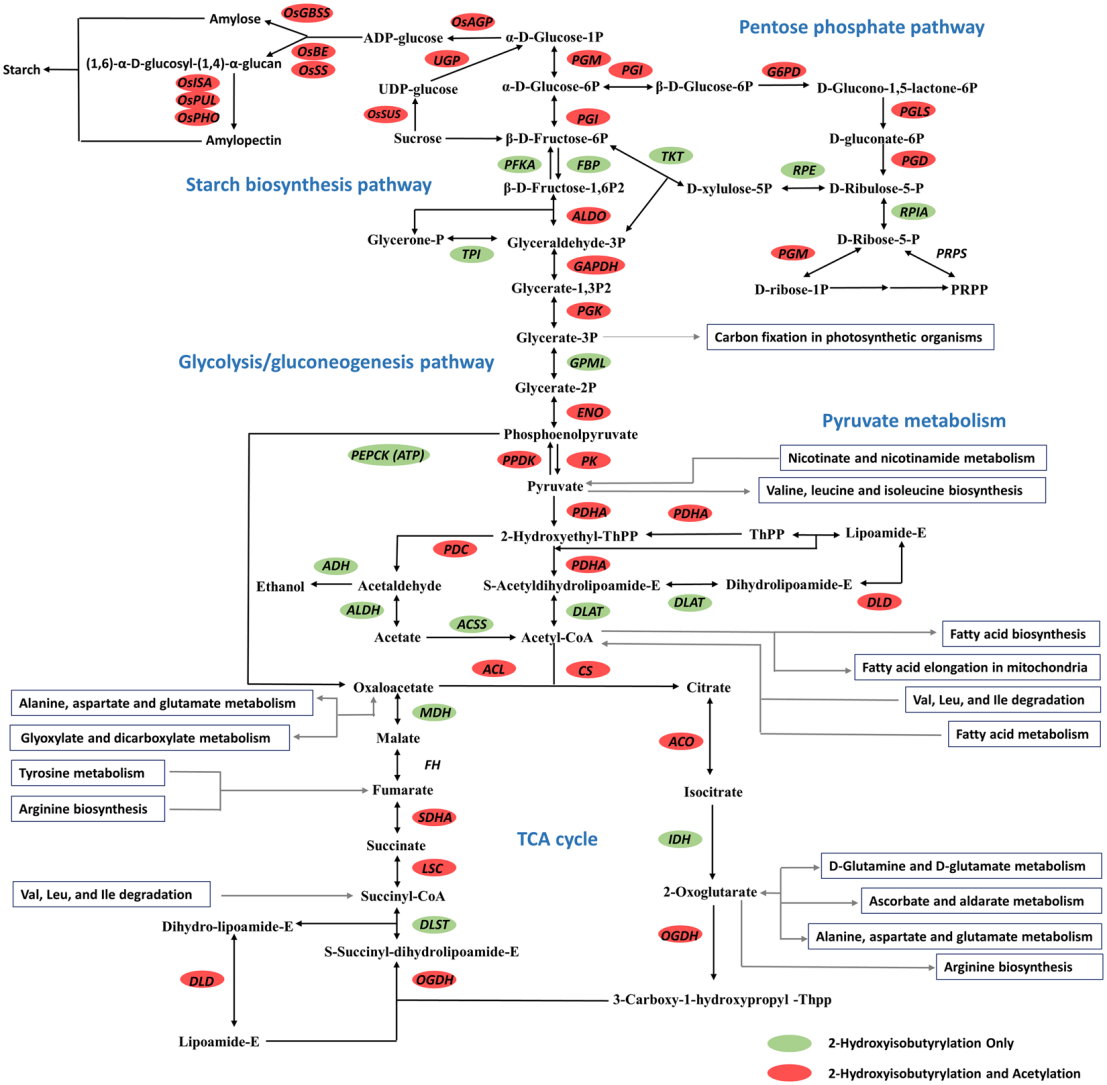
2-hydroxyisobutyrylated enzymes involved in TCA cycle, pentose phosphate pathway, pyruvate metabolism, glycolysis/gluconeogenesis and starch biosynthesis pathways. Proteins highlighted in green color are modified by 2-hydroxyisobutyrylation only, and proteins highlighted in red color are modified by both acetylation and 2-hydroxyisobutyrylation. The identified 2-hydroxyisobutyrylation sites and acetylation sites of enzymes in Fig. 4 are listed in Supplementary Table S3.

Metabolic pathway enrichment analysis using KEGG pathway annotation database demonstrated that proteins bearing K_hib_ were enriched in multiple central metabolic pathways including glycolysis/gluconeogenesis, TCA cycle, pyruvate metabolism, pentose phosphate pathway, and oxidative phosphorylation (Fig. 3b and Supplementary Table S2). We found almost every enzyme involved in glycolysis/gluconeogenesis, TCA cycle, pyruvate metabolism and pentose phosphate pathway were 2-hydroxyisobutyrylated (Fig. 4), suggesting a possible function of K_hib_ in regulating cellular glucose metabolism. A number of these enzymes were also acetylated (Fig. 4 and Supplementary Table S3). In addition to carbon metabolism, pathways related to protein biosynthesis and processing, including biosynthesis of amino acids, ribosome, protein processing in endoplasmic reticulum, and aminoacyl-tRNA biosynthesis were also enriched with 2-hydroxyisobutyrylated proteins (Fig. 3b). Notably, the 2-hydroxyisobutyrylome profiling of *S*.*cerevisiae* also revealed that the K_hib_ proteins were enriched in the glycolysis/gluconeogenesis pathway, ribosome, aminoacyl-tRNA biosynthesis pathway and in some amino acid metabolism pathways^8^, consistent with our KEGG pathway enrichment results.

Subcellular localization of 2-hydroxyisobutyrylated proteins in developing rice seeds was analyzed by WoLF PSORT software (Fig. 3c). The 2-hydroxyisobutyrylated proteins located in chloroplast and cytosol represented 36% and 31% of the total modified proteins, respectively, ranking as the top two classifications. Moreover, there were approximately 14%, 6%, and 5% 2-hydroxyisobutyrylated proteins assigned to the nucleus, mitochondria and plasma membrane, respectively.

### Storage proteins and proteins involve in starch and lipid metabolisms are preferably targeted by K_hib_

The enrichment analyses of GO annotation and KEGG pathway demonstrated that processes associated with biosynthesis of amino acid, translation factor, aminoacyl-tRNA ligase, ribosome, protein folding, protein transport and localization, were significantly enriched with 2-hydroxyisobutyrylation. Interestingly, other PTMs involving lysine were also significantly associated with protein biosynthesis and processing such as lysine malonylation in *E*.*coli*, lysine acetylation and succinylation in *M*. *tuberculosis* and *V*. *parahemolyticus* ^19–23^. These indicate a possible function of lysine modifications in coordinating protein biosynthesis and processing. In rice, we identified 10 glutelins and 3 globulins that were 2-hydroxyisobutyrylated (Supplementary Table S4). Storage proteins are predominant nutrition reservoir proteins in the rice grain endosperm during seed development and are important nitrogen source for seed germination and initial seedling development. Glutelin, accounting for 60–80% by weight of total seed protein, is the major seed storage protein in rice^24^. Out of the 15 glutelin storage proteins, 10 were 2-hydroxyisobutyrylated, including *GluA-1*, *GluA-2*, *GluA-3*, *GluB-1a*, *GluB-2*, *GluB-*5, *GluB-6*, *GluB-7*, *GluC-1*, and *GluD-1*. (Table S4). At least 50% of lysines were modified in 8 glutelin proteins including *GluA-1*, *GluA-3*, *GluB-1a*, *GluB-5*, *GluB-7*, *GluC-1*, *GluD-1* and globulin (Q75GX9) (Supplementary Table S4). *GluB-1a* showed the highest K_hib_ occupancy of 81.25% lysines among the modified storage proteins (Supplementary Table S4). These findings could indicate that lysine 2-hydroxyisobutyrylation may be involved in storage protein packaging, trafficking, or accumulation in the rice grain. However, further experiments are needed to prove these hypotheses. During digestion process, dietary proteins are broken down by digestive enzymes into peptides in the human body. Trypsin is one of the principal proteases in the human digestive system that cleaves peptide chain at the carboxyl side of lysine or arginine^25^. 2-hydroxyisobutyrylation blocks trypsin cleavage at lysine residues. The prevalence of 2-hydroxyisobutyrylation in rice grain storage proteins either via an enzymatic or nonenzymatic reaction may reduce the ability of trypsin to digest dietary proteins and nutrient absorption in human body.

A striking number of enzymes involved in starch biosynthesis were found to be modified by 2-hydroxyisobutyrylation including ADP-glucose pyrophosphorylases (*OsAGPS1*, *OsAGPS2*, *OsAGPL2* and *OsAGPL3*), granule-bound starch synthase I (*OsGBSSI*), starch branching enzymes (*OsBEI*, *OsBEIIa* and *OsBEIIb*), starch debranching enzymes (*OsISA3*, *OsISA2* and *OsPUL*), starch synthases (*OsSSI*, *OsSSIIa* and *OsSSIIIa*), starch phosphorylase (*OsPHOH* and *OsPHOL*), and disproportionating enzymes (*OsDPE1* and *OsDPE2*) (Fig. 4 and Supplementary Table S5). *OsAGPs* catalyze the first committed step of starch biosynthesis, which is related to rice grain filling rate and starch accumulation^26^. *OsGBSS* is responsible for synthesis of amylose and *OsSS*, *OsBE*, *OsISA*, *OsPUL* and *OsPHO* catalyze amylopectin formation. Starch quality regulators including *FLO2*, *FLO4*, and *Chalk5* were also 2-hydroxyisobutyrylated (see Supplementary Table S5). Mutation of *FLO2* and *FLO4* resulted in smaller grain size and floury-white endosperm^27,28^. Overexpression of *Chalk 5*, which regulates rice chalkiness, affects pH homeostasis of endomembrane trafficking system resulting in a chalky phenotype in the grain^29,30^. A study in *S*. *cerevisiae* revealed that K_hib_ on histone H4K8 is a glucose-responsive reaction through a pathway depending on glycolysis, and the K_hib_ proteome is closely related to glucose metabolism as well^8^. Connecting the study of *S*. *cerevisiae* with the fact that the essential enzymes for starch biosynthesis are 2-hydroxyisobutyrylated in rice, it is worthwhile to evaluate if K_hib_ plays a regulatory role in the starch synthesis during seed development.

Seed lipids have important nutritional roles during seed germination, seedling growth, and serve as an energy supplement for humans through dietary consumption^31,32^. In developing seeds, fatty acids are synthesized in the plastid with the initial input of acetyl-CoA from TCA cycle and beta-fatty acid oxidation. The resulting fatty acids are transported to the endoplasmic reticulum in the form of acyl-CoA. Rice seeds store lipids as triacylglycerol (TAG) which is synthesized from glycerol 3-phosphate and acyl-CoA by acyltransferases^31,33^. In this study, we found that lipid metabolism-associated proteins were 2-hydroxyisobutyrylated including acetyl-CoA C-acyltransferase, long chain acyl-CoA synthetase, 3-ketoacyl-CoA thiolase 2, 3-oxoacyl-[acyl-carrier-protein] synthase, acyl-coenzyme A oxidase, enoyl-CoA hydratase, enoyl-[acyl-carrier-protein] reductase, non-specific lipid transfer protein, acyl carrier protein, and lysophospholipase 2. In addition to K_hib_, proteins involved in lipid metabolism were also found modified by other lysine acylations such as succinylation and malonylation. In mouse liver, the majority of enzymes in the fatty acid oxidation were succinylated and a large proportion of the sites were overlapped with acetylation^34^. Proteins associated with fatty acid beta-oxidation and ketone body synthesis were significantly enriched with succinylation in mitochondria of mouse liver^35^. Increasing the rate of lysine malonylation resulted in impaired fatty acid oxidation in human cells^36^. It is possible that lipid metabolism is regulated by PTMs and K_hib_ may have a key role for lipid metabolism in cereal seeds as well.

### Lysine 2-hydroxyisobutyrylation of histone proteins in rice

**Figure 5:**
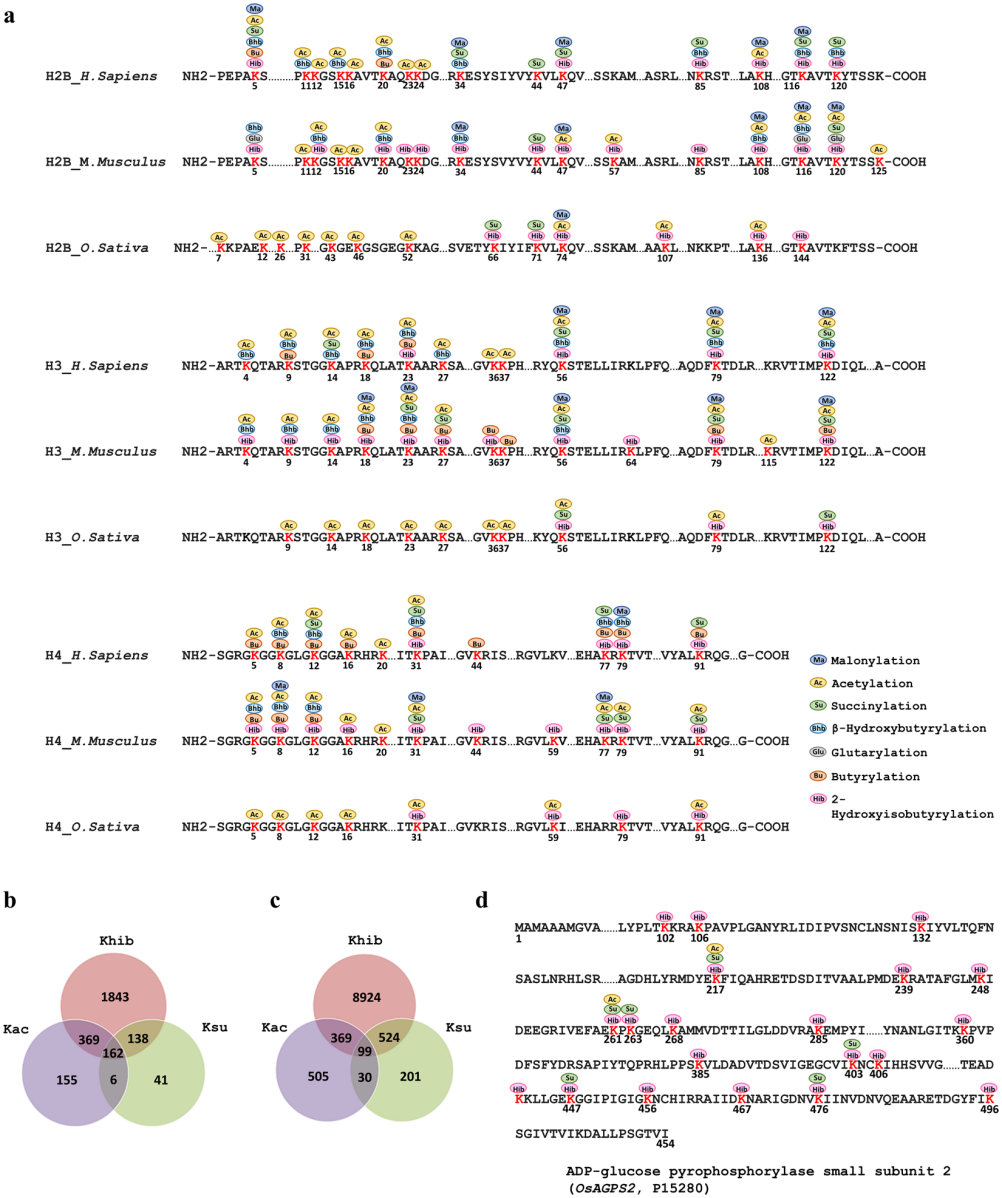
Conserveness of histone K_hib_ sites and overlapping among 2-hydroxyisobutyrylation and other PTMs. (**a**) Comparison of histone H2B, H3 and H4 lysine acylation sites among rice, human and mouse. Lysines with modifications are indicated by red color. The numbers indicate modified lysine positions on histones (**b**) Venn diagram showing the number of proteins overlapped among 2-hydroxyisobutyrylation, acetylation and succinylation in rice. (**c**) Venn diagram showing the number of lysine sites overlapped among 2-hydroxyisobutyrylation, acetylation and succinylation. (**d**) 2-hydroxyisobutyrylated, acetylated and succinylated sites on the representative protein ADP-glucose pyrophosphorylase small subunit 2 (P15280).

Emerging studies revealed that histone modifications and recognition genes are closely associated with many aspects of plant growth, development regulation, and responses to environmental conditions^37–41^. Affinity-directed HPLC/MS/MS analysis on histones identified 63 2-hydroxyisobutyrylation sites from HeLa cells and mouse total testis cells^3^. Unlike lysine acetylation, 2-hydroxyisobutyrylation is located in both N-terminal tail domains and other regions of core histones^3^. In this study, we identified 3 sites on histone H2A and 6 sites on H2B, while 3 and 4 sites on H3 and H4, respectively. Sequence alignment analysis found that rice 2-hydroxyisobutyrylation on H2BK71, H2BK74, H2BK136, H2BK144, H3K56, H3K79, H3K122, H4K31, H4K59, H4K79 and H4K91 were also observed in human and mouse (Fig. 5a), suggesting that lysine 2-hydroxyisobutyrylation is highly conserved across plants, human, and mouse. Meanwhile 2-hydroxyisobutyrylation on H2BK66 and H2BK107 were only identified in rice.

Apart from 2-hydroxyisobutyrylation, other types of histone acylations have been reported, including lysine acetylation, succinylation, malonylation, glutarylation, butyrylation, and β-hydroxybutyrylation^1,13,14,34,36,42–52^. An up-to date comprehensive list of these histone marks on human, mouse and rice are presented in Fig. 5a. Lysine 2-hydroxyisobutyrylation is a relatively abundant histone modification based on the number of histone marks in comparison with other PTMs. Figure 5a shows that a number of lysine residues on histones can be modified by multiple PTMs. Lysine 2-hydroxyisobutyrylation is favorable to form combinations with other lysine acylations on the same sites. For instance, acetylation, malonylation, succinylation, 2-hydroxyisobutyrylation and β-hydroxybutyrylation were detected on H3K56. Only one or two acylations have been identified in some histone lysine sites. H3k64 was detected to be unique to 2-hydroxyisobutyrylation. Only 2-hydroxyisobutyrylation and succinylation were identified on H2BK44, while H2BK23, 24, 57 and H4K59 harbor 2-hydroxyisobutyrylation and acetylation exclusively. The studies of glutarylation, butyrylation, and β-hydroxybutyrylation are lacking in rice, and data of other PTMs on rice was derived from proteome-wide acylation analyses instead of studies specific to histones. These explains that less histone PTMs were reported in rice in Fig. 5a.

### Comparison between K_hib_ and other PTMs in rice

We conducted a comparison of the current 2-hydroxyisobutyrylome dataset with lysine acetylome and lysine succinylome datasets identified in 15 dpa developing rice (*Oryza sativa* L. *japonica* cv. Nipponbare) seeds (ProteomeXchange dataset identifier PXD002773 and PXD005582) to investigate the similarities and differences among these lysine acyl modifications. The size of 2-hydroxyisobutyrylome reported in this study is significantly larger than the acetylome and succinylome identified in the same organ. We found that 531 and 300 2-hydroxyisobutyrylated proteins were also acetylated and succinylated, respectively (Fig. 5b and Supplementary Table S6). The three acyl modifications were overlapped in 162 proteins (Fig. 5b and Supplementary Table S6), which are mainly involved in central carbon metabolism, protein biosynthesis, energy production, signal transduction and storage proteins. Of the 2,512 identified proteins, 1,843 (73.37%) had only 2-hydroxyisobutyrylation detected compared to acetylation and succinylation (Fig. 5b). There were 468 and 623 2-hydroxyisobutyrylated lysine sites shared with acetylation and succinylation, respectively (Fig. 5c). Interestingly, starch biosynthesis rate-limiting enzyme ADP-glucose pyrophosphorylase small subunit 2 (*OsAGPS2*, P15280) was found heavily modified by 2-hydroxyisobutyrylation, acetylation, and succinylation. The modification sites of PTMs on *OsAGPS2* protein are highlighted in Fig. 5d. The co-modification of 2-hydroxyisobutyrylation, acetylation and succinylation on identical lysine sites in specific proteins suggests that these acyl modifications may share a common mechanism for modification site selection. It has been reported that p300 has enzymatic activities for lysine acetylation, succinylation and glutarylation^42,53^. It is unknown if similar proteins are involved in plants.

Lysine could allow multiple PTMs to occur at the same sites including acetylation, succinylation, propionylation, butyrylation, crotonylation, malonylation and glutarylation^5,34^. Crosstalks have been identified on acetylation of H3K9 and H3K27 with phosphorylation of Ser10 and Ser38, respectively^54,55^. It was also reported that downregulating succinylation levels by applying α-ketoglutarate dehydrogenase complex inhibitor CESP also diminished acetylation levels in cells^56^. Crosstalk between 2-hydroxyisobutyrylation and other PTMs may also exist and form a complex regulatory network on substrate functions and regulations. In this study, 99 sites were found modified by acetylation, succinylation and 2-hydroxyisobutyrylation, but the effect of the three different modifications on protein function remains to be investigated (Fig. 5c). A reported example of histone modifications may provide us with some clue. H4K8_hib_ and H4K8_ac_ are both related to active gene transcription, but H4K8_hib_ appeared to be a better indicator of transcriptional activity than H4K8_ac_^3^. However, the occurrence of specific PTMs and the interplay of functions among these PTMs are not yet clearly understood^5^.

## Conclusions

Lysine 2-hydroxyisobutyrylation is a protein post-translational modification initially identified on histones and associates with transcriptional activity regulation. In this study, we conducted a proteome-wide systematic mapping of lysine 2-hydroxyisobutyrylated sites in developing rice seeds. A total of 9,916 2-hydroxyisobutyryl lysine sites on 2,512 proteins were identified, which is significantly larger than the reported acetylome and succinylome in plants. Western blotting analysis indicated that lysine 2-hydroxyisobutyrylaton is a pervasive protein modification. Analysis of amino acid motifs at the modification sites revealed that negative charged amino acids, D and E, were strongly preferred around the 2-hydroxyisobutyrylated sites. Further analysis illustrated that 2-hydroxyisobutyryllysine is less surface accessible than unmodified lysine and it has a greater propensity to be found in regions of intrinsic disorder and coils. These structural features of 2-hydroxyisobutyrylation sites suggested that the modification occurs in a highly selective process. GO and KEGG pathway enrichment analyses showed that 2-hydroxyisobutyrylated proteins were involved in multiple important biological processes including central carbon metabolisms, protein biosynthesis and processing. The widespread 2-hydroxyisobutyrylation in rice grain storage proteins was observed and may have an impact on the ability of trypsin to digest crop proteins in human body. Moreover, proteins associated with starch biosynthesis pathway and lipid metabolism were also favorably targeted by 2-hydroxyisobutyrylation. The conserved K_hib_ sites on histone proteins were identified across human, mouse and rice proteomes. Through comprehensive comparison of rice acetylome, succinylome and 2-hydroxyisobutyrylome, we found that 99 2-hydroxyisobutyrylated sites could be modified by any of the three PTMs, while 8924 sites were unique to K_hib_, indicating that these acylations may have distinct but also overlapping regulations. As the first proteome-wide study of lysine 2-hydroxyisobutyrylation in plants, our results provide novel insight of protein 2-hydroxyisobutyrylation on non-histone proteins.

## Materials and Methods

### Plant materials and growth conditions

Rice plants of *Oryza sativa* L. *japonica* cv. Nipponbare were used in this study. The leaves and roots of 20-day seedlings grown in incubator at 28 °C (16-h-day/8-h-night) were sampled. The flowers, pollens, 7, 15 and 21 days post anthesis (dpa) developing rice seeds and mature rice seeds were collected from rice plants grown at 30 °C during day and 25 °C at night in greenhouse of Mississippi State University, MS, USA. The cultured cells are rice (*Oryza sativa* L. *japonica* cv. Nipponbare) NB2P suspension cell, which were maintained as reported^57,58^.

### Protein extraction, western blotting and proteolytic enzyme digestion

Proteins were extracted by phenol isolation method as previously reported^59–61^. For western blotting, 2-hydroxyisobutyrylated proteins were detected by using rabbit-derived pan anti-2-hydroxyisobutyryllysine antibody (PTM-801, PTM Biolabs, Chicago, IL, USA) in a 1:1000 (v/v) dilution overnight at 4 °C with gentle shaking as suggested by the supplier. Anti-rabbit IgG alkaline phosphatase conjugated secondary antibody was used at a 1:30,000 (v/v) dilution.

Before digestion, proteins were dissolved in 8 M urea, 100 mM NH_4_HCO_3_ (pH 8.0), reduced with 10 mM Dithiothreitol for 1 h at 37 °C and subsequently alkylated with 20 mM Iodoacetamide for 45 min at room temperature in dark. For proteolytic enzyme digestion, the protein sample was diluted to reduce the urea concentration to less than 2 M by adding 100 mM NH_4_HCO_3_. Then, sequencing grade trypsin (V5111, Promega Corporation, Madison, WI, USA) was added at a 1:50 (w/w) enzyme-to-substrate mass ratio for overnight digestion and 1:100 (w/w) enzyme-to-substrate mass ratio for additional 4 h-digestion at 37 °C.

### HPLC fractionation and affinity enrichment of peptides

To reduce the sample complexity, high-pH reverse-phase HPLC with Agilent 300 Extend C18 column (5 μm particles, 4.6 mm ID, 250 mm length, Agilent, Santa Clara, CA, USA) was used to separate proteolytic peptides before enrichment of 2-hydroxyisobutyrylated peptides. In short, peptides were first separated into 80 fractions with a gradient of 2% to 60% acetonitrile (ACN) in 10 mM (NH_4_)HCO_3_, pH 10.0. Afterwards, the peptides were combined into 8 fractions in a noncontiguous manner as reported^62^ and dried under vacuum. For K_hib_ peptides enrichment, fractionated peptides were re-dissolved in NETN buffer consisting of 100 mM NaCl, 1 mM EDTA, 50 mM Tris-HCl, 0.5% NP-40, pH 8.0 and, incubated with pre-washed pan anti-2-hydroxyisobutyryllysine antibody conjugated agarose beads (PTM-804, PTM Biolabs, Chicago, IL, USA) at 4 °C overnight with gentle oscillation. Then, the beads were rinsed four times with NETN buffer and twice with ice cold sterilized ddH_2_O. Enriched peptides were eluted by 0.1% Trifluoroacetic acid from beads and cleaned up with C18 ZipTips column (EMD Millipore, Billerica, MA, USA).

### Mass spectrometric analysis

The peptides were dissolved in 0.1% formic acid (FA) and loaded onto a reversed-phase pre-column (Acclaim PepMap 100 C18 column, 2 μm particles, 75 μm ID, 2 cm length, Thermo Fisher Scientific, Waltham, MA, USA), followed by peptide separation using a reversed-phase analytical column (Acclaim PepMap RSLC, 2 μm particles, 50 μm ID, 15 cm length, Thermo Scientific, Waltham, MA, USA). The solvents were delivered at a constant flow rate of 280 nl/min by a nanoflow HPLC pumps of EASY-nLC 1000 UPLC system (Thermo Scientific, Waltham, MA, USA). The analytical gradient was from 7% to 18% of solvent buffer consisting of 0.1% FA in 98% ACN in 16 min, followed by an increased concentration from 18% to 22% in 8 min, and then 22% to 35% in another 8 min, further climbing to 80% in 5 min and stay at 80% of the solvent buffer for the last 3 min. The resulting peptides were subjected to a NanoSpray Ionization (NSI) source followed by Q-Exactive Plus Hybrid Quadrupole-Orbitrap mass spectrometer (Thermo Fisher Scientific, Waltham, MA, USA) analysis. Intact peptides were detected at a resolution of 70,000 with scan range of 350–1800 m/z for full MS scans in the Orbitrap. Peptides were selected for MS/MS using NCE setting as 30%. Ion fragments were detected at a resolution of 17,500. And a data-dependent procedure that alternated between one MS scan followed by 20 MS/MS scans was applied for the top 20 precursor ions above a threshold ion count of 1.0 × 10^4^ in the MS survey scan with 15.0 s dynamic exclusion. The electrospray voltage applied was 2.0 kV and automatic gain control (AGC) was used to prevent overfilling of the ion trap. Ions with charge state of 2 to 5 were allowed and 5 × 10^4^ ions were accumulated for generation of MS/MS spectra.

### Database searching

The MS/MS raw data was processed using MaxQuant (http://www.maxquant.org/) with integrated Andromeda search engine (v.1.4.1.2). Database for *Oryza sativa* used was obtained from Uniprot containing 63,195 sequences (released July 2014). It also concatenates with reverse database and common contaminants. Precursor and fragment ions mass tolerances were set to 10 ppm and 0.02 Da, respectively. Trypsin/P was specified as cleavage enzyme, allowing a maximum of 4 missing tryptic cleavages, 5 modifications per peptide and 5 charges. Static modification used was Carbamidomethylation for cysteine. Dynamic modifications included oxidation on methionine and 2-hydroxyisobutyrylation on both lysine and protein N-terminal. Minimum peptide length was set at 7. The false discovery rate (FDR) thresholds for protein, peptide and modification site were set at 1%. Lysine 2-hydroxyisobutyrylation sites identified with a localization probability of <0.75 were removed.

### Bioinformatics analysis of 2-hydroxyisobutyrylated peptides and proteins

Gene ontology (GO) analysis was derived from the UniProt-GOA database (http://www.ebi.ac.uk/GOA/) based on the categories of biological process and molecular function. Proteins that were not annotated by UniProt-GOA database were further searched by InterProScan software according to protein sequence alignment. Kyoto Encyclopedia of Genes and Genomes (KEGG) pathway analysis was performed by KEGG Automatic Annotation Server (KAAS) tool, and the annotation results were mapped to KEGG pathway database by using online service tool KEGG mapper. Additionally, GO and KEGG pathway enrichment analyses were carried out by functional annotation tool of DAVID bioinformatics resources 6.7 against the background of rice (*Oryza Sativa*). A two-tailed Fisher’s exact test was used to check the identified 2-hydroxyisobutyrylated proteins. Correction for multiple hypothesis testing was carried out using standard FDR control method and annotation terms with a corrected p-value below 0.05 were considered as significantly enriched. The subcellular localization of identified proteins were predicated by WoLF PSORT software.

Sequence model around 2-hydroxyisobutyryl lysine was analyzed by motif-x (http://www.motif-x.med.harvard.edu) with ten amino acids upstream and downstream of the modification sites. Protein sequences of all proteins of *Oryza sativa L*. *japonica* were used as background database. Cluster membership was visualized by heat map through the “heatmap.2” function in the “gplots” R-package. Secondary structure analysis was performed using NetSurfP (version 1.1). Only predictions with a minimum probability of 0.5 for one of the different secondary structures (coil, α-helix, β-strand) were considered for analysis. The mean secondary structure probabilities of modified lysine residues were compared with the mean secondary structure probabilities of a control dataset containing all lysine residues of all modified proteins identified in this study. *p* values were calculated using non-paired Wilcox test.

### Data Availability

The mass spectrometry proteomics data have been uploaded onto the ProteomeXchange Consortium via the PRIDE partner repository with the dataset identifier of PXD005986.

## Electronic supplementary material

Supplementary Dataset 1
